# Fate of pulmonary hypertension associated with bronchopulmonary dysplasia beyond 36 weeks postmenstrual age

**DOI:** 10.1136/archdischild-2019-318531

**Published:** 2020-06-22

**Authors:** Sanne Arjaans, Meindina G Haarman, Marcus T R Roofthooft, Marian W F Fries, Elisabeth M W Kooi, Arend F Bos, Rolf M F Berger

**Affiliations:** 1 Pediatric and Congenital Cardiology, University Medical Centre Groningen, University of Groningen, Groningen, The Netherlands; 2 Division of Neonatology, University Medical Centre Groningen, University of Groningen, Groningen, The Netherlands

**Keywords:** neonatology, cardiology

## Abstract

**Objective:**

To determine the survival and evolution of pulmonary hypertension (PH) associated with bronchopulmonary dysplasia (BPD) in extremely premature born infants beyond 36 weeks postmenstrual age (PMA).

**Design:**

A single-centre retrospective cohort study from a university hospital.

**Patients:**

Extremely preterm (gestational age <30 weeks and/or birth weight <1000 g) infants, born between 2012 and 2017, in the University Medical Center Groningen with confirmed PH at/beyond 36 weeks PMA.

**Main outcome measures:**

Survival, mortality rate and PH resolution. Patient characteristics, treatment, presence and evolution of PH were collected from patient charts.

**Results:**

Twenty-eight infants were included. All had BPD, while 23 (82%) had severe BPD and 11 infants (39%) died. Survival rates at 1, 3 and 7 months from 36 weeks PMA were 89%, 70% and 58%, respectively. In 16 of the 17 surviving infants, PH resolved over time, with a resolution rate at 1 and 2 years corrected age of 47% and 79%, respectively. At 2.5 years corrected age, the resolution rate was 94%.

**Conclusions:**

These extremely preterm born infants with PH-BPD had a survival rate of 58% at 6 months corrected age. Suprasystemic pulmonary artery pressure was associated with poor outcome. In the current study, infants surviving beyond the corrected age of 6 months showed excellent survival and resolution of PH in almost all cases. Prospective follow-up studies should investigate whether resolution of PH in these infants can be improved by multi-modal therapies, including respiratory, nutritional and cardiovascular treatments.

**What is already known on this topic?**

- Due to advances in perinatal care, survival of extremely low-gestational-age neonates beyond 36 weeks postmenstrual age has been improved.
- There is an increased recognition that pulmonary hypertension complicates the prognosis of extremely preterm infants with bronchopulmonary dysplasia beyond 36 weeks postmenstrual age.
- According to recent meta-analyses, there are insufficient data on the longer-term outcome of premature born infants with pulmonary hypertension and bronchopulmonary dysplasia (PH-BPD).

**What this study adds?**

- This study determined that the survival rate of extremely preterm infants with BPD-PH at 6 months corrected age was 58%.
- In extremely preterm infants with PH-BPD who survived beyond a corrected age of 6 months, PH had resolved at 2.5 years of follow-up in most of the cases.

## Introduction

Advances in perinatal medicine have resulted in improved survival of extremely preterm born infants, but there still remains increased risk of respiratory morbidity, including the development of bronchopulmonary dysplasia (BPD), and mortality following preterm birth.^1 2^ Pulmonary vascular disease and late pulmonary hypertension (PH) are commonly recognised associations of BPD in preterm infants.^3–7^ PH develops most frequently in infants with severe BPD, is associated with significantly increased mortality and is recently suggested to be associated with significant pulmonary morbidity in long-term survivors.^8–10^

A recent systematic review and meta-analysis estimated 20% of the infants with BPD developed PH, with reported prevalence ranging from 3% to 44% correlating with the severity of BPD.^8^ The highest rates of PH were reported in infants with severe BPD.^7 8 11–14^ Only 2% of infants without BPD were estimated to develop PH.^8^ Reported mortality rates in extremely preterm infants with PH ranged from 14% to 38%, although heterogeneity in study design hamper generalisability of these mortality rates.^7–13 15^ Meta-analysis estimated a mortality rate before discharge of 16% (95% CI 7% to 28%), whereas mortality rate at longer follow-up was estimated at 40% (95% CI 26% to 54%).^8^ However, available data on survival of premature infants with PH beyond 36 weeks postmenstrual age (PMA) are scarce.^9 10^

There has been a recent focus on risk assessment, evaluation and management of late PH in premature infants with BPD during the neonatal period,^15 16^ but the clinical course and long-term outcomes in this high-risk population beyond 36 weeks PMA remains insufficiently characterised. Resolving this knowledge gap could support development of guidelines for screening and monitoring extremely preterm born infants at risk for PH.^8 15^ Accordingly, this study aims to describe the long-term clinical course of extremely preterm born infants with PH-BPD beyond 36 weeks PMA, including mortality rate and the evolution of PH in surviving infants. The secondary aim is to identify clinical variables associated with survival in extremely premature born infants with PH-BPD.

## Methods

### Study population

Infants with a gestational age <30 weeks and/or birth weight <1000 g, born in the University Medical Center Groningen between 1 January 2012 and 31 December 2017, in whom PH-BPD was echocardiographically confirmed at or beyond 36 weeks PMA were included in the study. In the absence of a standardised screening protocol for PH in extremely premature infants with BPD during the study period, the diagnosis of PH-BPD was made by echocardiographic evaluation performed at the discretion of the treating physician.

### Data collection

Demographic, maternal and neonatal data and, if applicable, time of death were collected retrospectively from the Dutch National registry for PH in Childhood (Med Ethic no. M11.097816). Data were collected until February 2019 and stored using a REDCap (Research Electronic Data Capture) database.^17^

In this preterm born infant population, PH was defined as the presence of one or more of the echocardiographic findings described in table 1.^7 18^ For the current study, PH had to be confirmed echocardiographically at or beyond 36 weeks PMA in the absence of signs of LV dysfunction and/or pulmonary vein stenosis.

**Table 1:** Pulmonary hypertension (PH) definition

PH	In the presence of a (supra)cardiac shunt
	Bi-directional or R-L flow through shunt
	In the absence of a (supra)cardiac shunt
	RVSP >40 mm Hg and/or
	RVSP/SBP >0.5 and/or
	Every degree of systolic septal flattening

RVSP, right ventricular systolic pressure; SBP, systolic blood pressure.

The presence and severity of BPD were assessed at 36 weeks PMA using the NIH standard for definition and grading of BPD.^5^ At 36 weeks PMA, BPD was diagnosed if the infant had received supplemental oxygen (>21%) for at least 28 days; *mild BPD*, when an infant did not receive supplemental oxygen anymore at 36 weeks PMA; *moderate BPD*, when an infant received supplemental oxygen below 30% at 36 weeks PMA; and *severe BPD*, when an infant received more than 30% supplemental oxygen at 36 weeks PMA.

### Outcome parameters

Predefined outcome parameters were mortality and resolution of PH-BPD. The date of resolution of PH was defined as the date of first echocardiograph showing no signs of PH anymore, if available, confirmed by subsequent echocardiograms without signs of PH.

### Statistical analyses

Data are presented, depending on the distribution of the variable, as mean±SD, median (IQR) or frequencies with percentages. R and R studio V.1.14 statistical software was used for statistical analyses.^19 20^ All tests were two sided and p values <0.05 considered significant. Characteristics of survivors and non-survivors were compared using Student’s t-test, Mann-Whitney U test or χ^2^ test as appropriate. To determine variables associated with survival (time dependent), univariate Cox regression analyses were performed. Kaplan-Meier curves were computed to depict survival of patient groups and log rank test to compare groups.

## Results

### Demographic and clinical characteristics

Twenty-eight infants with PH diagnosed by echocardiography at or beyond 36 weeks PMA were included. Two infants showed increased flow velocities with biphasic flow pattern in one pulmonary vein, not regarded clinically significant pulmonary vein stenosis. Only two infants underwent cardiac catheterisation confirming the diagnosis of PH. Eighteen infants (64%) were male. The median gestational age was 26.4 weeks (IQR 25.6–27.6), mean birth weight 790.0±234.2 g. All 28 infants were diagnosed with BPD at 36 weeks PMA, mostly with severe BPD (n=23, 82%). Six infants (21%) had suffered from necrotising enterocolitis, nine infants (32%) from interventricular haemorrhage and nine infants (32%) from retinopathy of prematurity. Patient characteristics of included infants are shown in table 2. In the absence of a standardised treatment regimen for PH-BPD, 14 infants (50%) received pulmonary arterial hypertension (PAH)–targeted therapy at the discretion of the treating physician. All 14 received sildenafil: 11 (79%) as monotherapy and 3 (21%) as part of dual therapy (two infants in combination with oral bosentan and one in combination with subcutaneous treprostinil). Nine infants received inhaled nitric oxide (iNO) treatment: three infants received iNO before 36 weeks PMA, one of these also beyond 36 weeks PMA and six other infants received iNO only beyond 36 weeks PMA (table 2). The median follow-up of the infants was 2.75 (IQR 1.2–3.9) years.

**Table 2:** Patient characteristics

Patient characteristics	All infants n=28	N	Survivors n=17	N	Non-survivors n=11	N	P value
Before 36 weeks PMA							
Male sex, n (%)	18 (64)	28	11 (65)	17	7 (64)	11	1.00
Gestational age, weeks	26.4±1.6	28	26.4±1.4	17	26.4±1.8	11	0.40
Birth weight, g	790.0±234.2	28	820.6±267.8	17	742.7±171.2	11	0.36
SGA, n (%)	8 (29)	28	5 (29)	17	3 (27)	11	1.00
PPROM, n (%)	5 (19)	27	3 (19)	16	2 (18)	11	1.00
Pre-eclampsia, n (%)	9 (33)	27	5 (31)	16	4 (36)	11	1.00
Multigestation, n (%)	2 (7)	27	1 (6)	16	1 (9)	11	1.00
Presence of NEC, n (%)	6 (21)	28	1 (6)	17	5 (45)	11	**0.02**
Presence of IVH, n (%)	9 (32)	28	5 (29)	17	4 (36)	11	1.00
Grade I	4 (14%)	28	2 (12%)	17	2 (18%)	11	0.71
Grade II	4 (14%)	28	3 (18%)	17	1 (9%)	11	
Grade III	1 (4%)	28	0 (0%)	17	1 (9%)	11	
Grade IV	0 (0%)	28	0 (0%)	17	0 (0%)	11	NA
Presence of ROP, n (%)	9 (32)	28	6 (35)	17	3 (27)	11	1.00
Received dexamethasone, n (%)	6 (21)	28	3 (18)	17	3 (27)	11	1.00
Received iNO therapy, n (%)	3 (11)	28	2 (12)	17	1 (9)	11	0.22
At and beyond 36 weeks PMA							
Respiratory status at 36 weeks PMA							**0.02**
Ventilator, n (%)	0 (0)	28	0 (0)	17	0 (0)	11	
CPAP, n (%)	8 (29)	28	2 (12)	17	6 (55)	11	
Supplemental O_2_, n (%)	15 (54)	28	10 (59)	17	5 (45)	11	
BPD, n (%)	28 (100)	28	17 (100)	17	11 (100)	11	0.56
Mild BPD, n (%)	4 (14)	28	3 (18)	17	1 (9)	11	
Moderate BPD, n (%)	1 (4)	28	1 (6)	17	0 (0)	11	
Severe BPD, n (%)	23 (82)	28	13 (76)	17	10 (91)	11	
Age at PH confirmation beyond 36 weeks PMA, months	2.5 (1.6–4.6)	28	2.7 (1.7–5.4)	17	2.1 (0.4–3.1)	11	0.12
Suprasystemic pressures, n (%)	9 (32)	28	3 (18)	17	6 (55)	11	0.095
PDA, n (%)	21 (84)	25	11 (73)	15	10 (91)	11	0.36
PAH-targeted therapy, n (%)	14 (50)	28	10 (59)	17	4 (36)	11	0.44
Monotherapy, n (%)	11 (39)	28	9 (53)	17	2 (18)	11	0.12
Dual therapy, n (%)	3 (11)	28	1 (6)	17	2 (18)	11	0.54
Dexamethasone, n (%)	7 (25)	28	4 (24)	17	3 (27)	11	1.00
iNO therapy, n (%)	7 (25)	28	4 (35)	17	3 (27)	11	0.22
PH resolved, n (%)	16 (57)	28	16 (94%)	17	0 (0%)	11	**<0.0001**

Comparisons between survivors and non-survivors were performed with Student’s t-test, Mann-Whitney U test and χ^2^ test, used accordingly. The p value shown is from the comparison between the survivors and non-survivors. Data are shown as N (%), mean±SD or median (IQR).

BPD, bronchopulmonary dysplasia; CPAP, continuous positive airway pressure; iNO, inhaled nitric oxide; IVH, any grade of interventricular haemorrhage; NA, not available; NEC, necrotising enterocolitis; PAH, pulmonary arterial hypertension; PDA, patent ductus arteriosus; PFO, patent foramen ovale; PH, pulmonary hypertension; PMA, postmenstrual age; PPROM, prolonged premature rupture of membranes (>24 hours ruptured membranes before <36 weeks gestational age); ROP, any grade of retinopathy of prematurity; SGA, small for gestational age.

### Outcome

**Figure 1:**
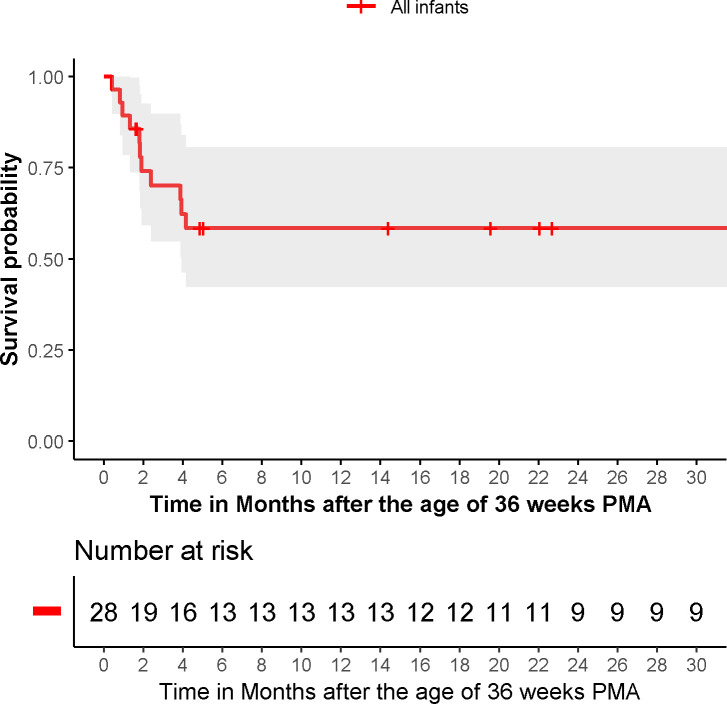
Survival rate of all the 28 included infants is shown. The shading represents the 95% CI. In addition, the number of infants is shown. PMA, postmenstrual age.

**Figure 2:**
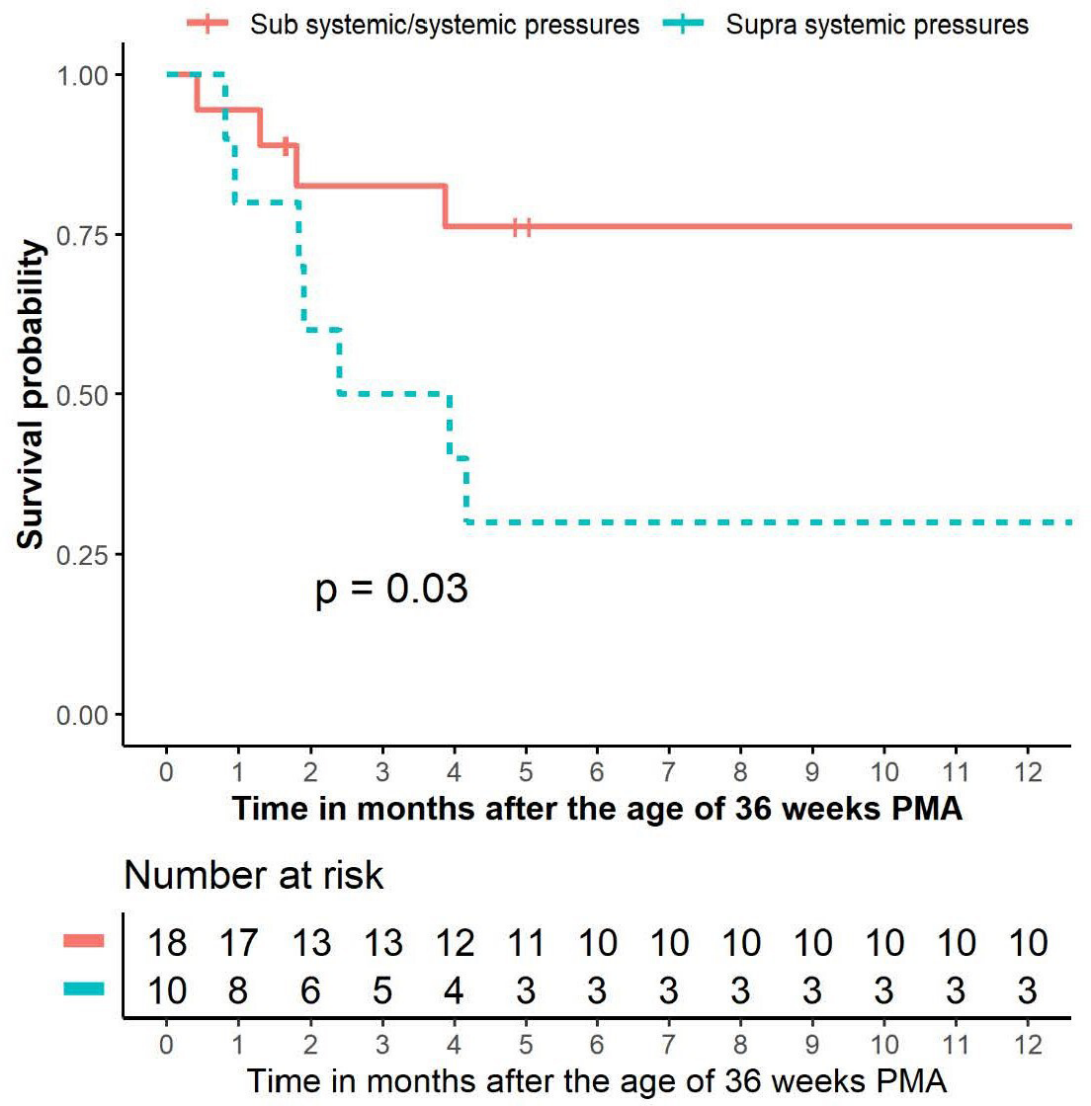
Survival rate of all the 28 included infants, divided into pulmonary hypertension with subsystemic/systemic vs suprasystemic pulmonary arterial pressure. In addition, the number of infants is shown. A log-rank test was used for the comparison between the two groups. PMA, postmenstrual age.

Eleven infants (39%) died during follow-up, attributed to the combination of BPD and PH in eight infants, to BPD in two and to sepsis in one. Survival rates at 1, 3 and 7 months after 36 weeks PMA were 89%, 70% and 58%, respectively (figure 1). Table 2 shows comparisons of patient characteristics between survivors and non-survivors. Infants who died had suffered more often from NEC. No further differences in neonatal and maternal parameters were observed between survivors and non-survivors. Time-dependent univariate Cox regression analysis revealed the presence of suprasystemic pulmonary artery pressure (PAP) to be associated with worse survival (HR 6.46, 95% CI 1.86 to 22.44; p=0.003), also when adjusted for gestational age (HR 6.40, CI 5.02 to 8.16; p<0.001). Univariate cox regression analyses showed that continuous positive airway pressure (CPAP) dependency at 36 weeks PMA was almost significantly associated with a worse survival (HR 3.27, 0.99 to 10.73; p=0.05). Univariate cox regression analyses could not identify other variables to be correlated with survival: gestational age (HR 0.77, 0.49 to 1.20; p=0.24), SGA (HR 0.87, 0.23 to 3.28; p=0.83), BPD severity (HR 2.15, 0.27 to 16.86; p=0.47) and receiving any type of PAH-targeted therapy (HR 0.99, 0.27 to 3.57; p=0.98). Kaplan-Meier curve shows worse survival of infants with suprasystemic PAP (1-month, 3-month and 7-month survival rates of 80%, 50% and 30%, respectively) compared with those with (sub)systemic PAP (1-month, 3-month and 7-month survival rates of 94%, 83% and 76%, respectively; log-rank test, p=0.03) (figure 2).

**Figure 3:**
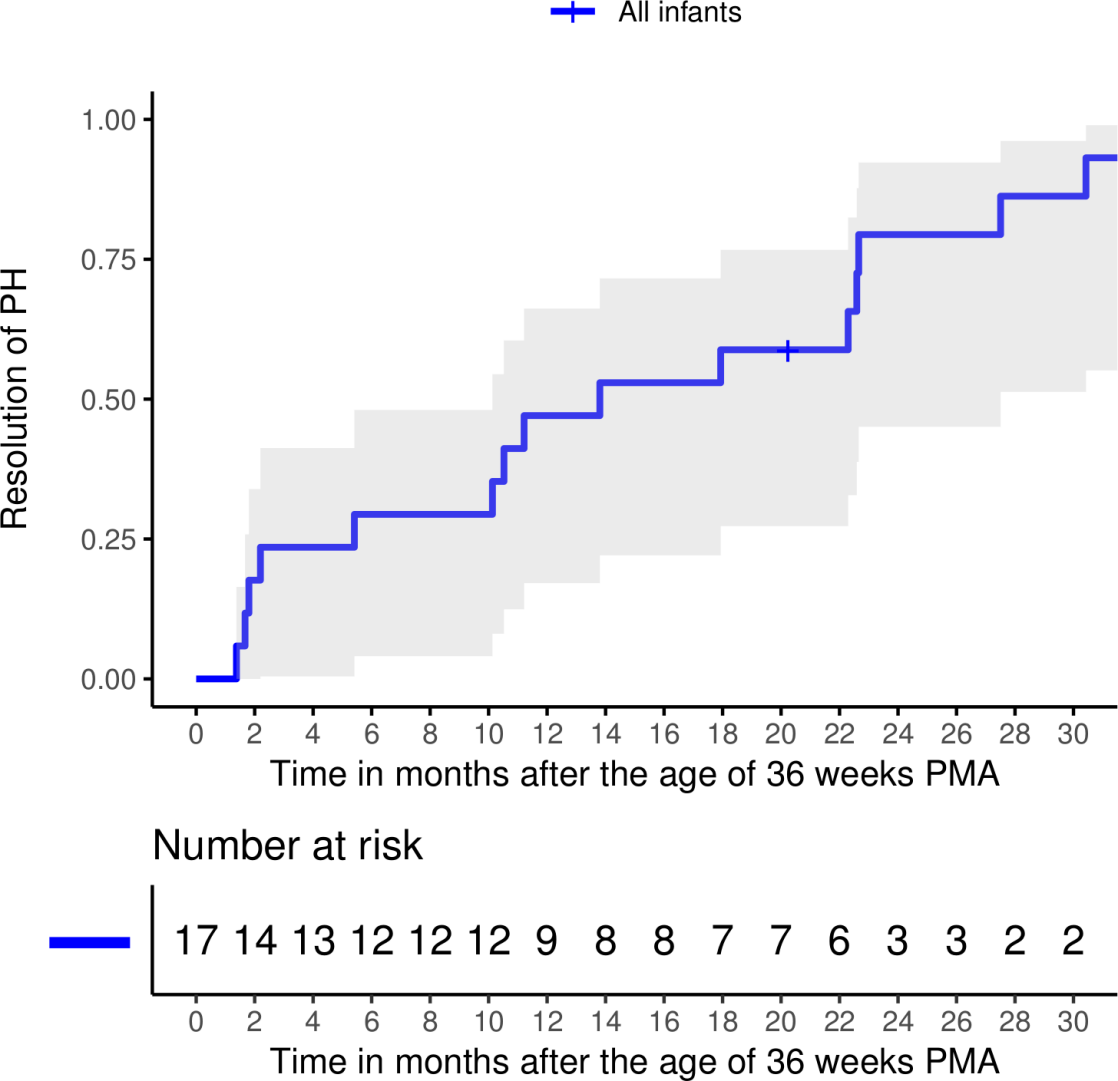
Resolution rate during the follow-up of the 17 surviving infants is shown. The shading represents the 95% CI. In addition, the number of infants is shown. PH, pulmonary hypertension; PMA, postmenstrual age age.

**Figure 4:**
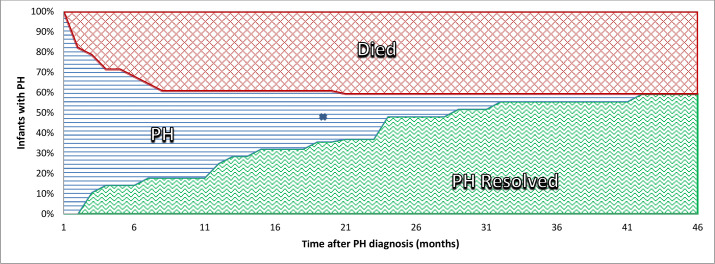
Number of infants with pulmonary hypertension (PH) and the mortality and resolution rate of the included population are shown. X=infant censored due to loss to follow-up.

In the surviving infants (n=17), PH appeared to resolve during follow-up with resolution rates at 1 and 2 years after 36 weeks PMA of 47% and 79%, respectively (figure 3). At 2.5 years after 36 weeks PMA, the resolution rate had increased to 94%. One infant was lost to follow-up. Figure 4 shows an overview of mortality and PH resolution during follow-up of infants with PH-BPD diagnosis at or beyond 36 weeks PMA.

## Discussion

This study reports long-term survival of extremely preterm infants with PH-BPD present at or beyond 36 weeks PMA and found a low survival rate of less than 60% at a corrected age of 6 months. Mortality occurred predominantly within the first half year after reaching 36 weeks PMA. Notably, almost no further mortality occurred after this period during a median follow-up of 2.8 (1.2–3.9) years. In surviving infants PH resolved over time with a resolution rate up to almost 100% in 2.5 years. The observed survival rate in the current study is in line with the scarce data from these previous reports, with survival ranging from 53% to 74%.^9 10 21^ Our study adds to the existing literature by providing data on timing of mortality, showing a favourable prognosis regarding survival with resolution of PH in infants surviving 6 months after corrected term age.

In the current study, demographical characteristics did not differ between infants who died during follow-up and those who did not. However, the severity of PH and respiratory status at 36 weeks PMA appeared predictive for outcome in PH-BPD. Suprasystemic PAP and CPAP dependency at 36 weeks PMA were associated with higher mortality rate. These observations are supported by previous observations. Altit *et al* studied infants ≤32 weeks gestational age with PH at 36 weeks PMA and identified PH severity and postnatal steroid use as risk factors for death.^22^ Recently, Lagatta and colleagues retrospectively studied mortality through 1 year after 36 weeks PMA in a large cohort of 370 preterm infants with PH-BPD retrieved from the Children’s Hospital Neonatal Database and found ventilator dependency at 36 weeks PMA, postnatal steroids and the presence of atrial septal defect associated with mortality in the first year.^21^

In the current study, all infants, except one, that survived the first 7 months of follow-up showed resolution of PH over time. One infant was lost to follow-up. Although resolution of PH in infants with BPD has been reported incidentally, the current study had a long and standardised follow-up in all infants and found a substantial higher resolution rate.^9 10 22 23^ Del Cerro observed in a selected group of infants with PH-BPD, referred to a tertiary PH unit, that PH ‘resolved or improved’ in 17 of 21 surviving infants at median follow-up of 35 months, although PH recurred in two infants after withdrawal of sildenafil. Few other studies report PH resolution in the first year after PH diagnosis. Discrepancies in resolution rates are likely explained by heterogeneity in patient characteristics in different cohorts of highly selected infants. One instance is gestational age: very preterm infants, ≤24 weeks gestational age, have most pronounced pulmonary vascular and airway immaturity and injury, probably associated with more severe and longer lasting PH. Management approaches to such very preterm infants differ between countries. In the Netherlands, active ventilator treatment is offered generally only to infants born ≥24 weeks, whereas in other countries infants born <24 weeks do receive active treatment. The resolution rate in the current study suggests that infants born at ≥24 weeks surviving the first 6 months after corrected age have a high chance of long-term survival and resolution of PH in the first 3 years after birth. However, PH resolution is not synonymous to normalisation of the pulmonary vasculature. Evidence is emerging indicating that extremely preterm born infants with early pulmonary vascular disease are still at risk for cardiovascular and respiratory morbidity later in life.^24–27^ Late respiratory morbidity includes asthma, reactive airways disease, BPD exacerbation, bronchiolitis, pneumonia or respiratory-related hospitalisation in the first years of life.^27^ Mildly increased PAP, stiffer pulmonary vasculature, right ventricular dysfunction and impaired ventriculo-vascular coupling have been suggested in young adults born prematurely.^24–26^ These observations imply that infants who experience a resolution of their PH deserve a regular follow-up for signs of cardiovascular disease, including PH. Prospective studies are now needed to accurately assess the evolution of PH and the clinical burden of PH in these former preterm born infants with BPD.

Diagnosis and classification of PH in preterm infants with or without BPD is challenging. One strength of the current study is the use of a standardised echocardiographic definition for the diagnosis of PH. Nevertheless, echocardiography for the diagnosis of PH has intrinsic limitations, including the estimation of PAP derived from indirect measurements instead of actual pressure measurements, the difficulty to exclude left ventricular (diastolic) dysfunction and pulmonary vein stenosis often associated with compromised echo windows in infants with chronic lung disease and finally the operator dependence of the technique. Several emerging echocardiographic variables have been suggested to improve accuracy of echocardiographic PH diagnosis, but also to enhance its value in monitoring disease severity and predicting outcome in this specific infant population.^18 28 29^ These include pulmonary artery acceleration time and tricuspid annular plane systolic excursion. Gold standard for diagnosing PH remains cardiac catheterisation, but in the vulnerable population of extremely preterm infants, such invasive procedure with associated risks is quite controversial. The optimal approach to diagnosis and monitoring of pulmonary hypertension in preterm born infants remains unclear. Recently, a report of a dedicated PPHNet workshop summarised the challenges of obtaining comprehensive information regarding disease severity and the relative contributions of pulmonary vascular disease, cardiac dysfunction and lung disease in preterm infants.^16^ According to the current World Symposium on Pulmonary Hypertension clinical classification of PH, PH-BPD is classified as group 3 associated with developmental lung diseases, but concomitant comorbidities such as cardiac shunts, collaterals and pulmonary vein stenosis may obscure the type of PH.^30^ The use of PAH-targeted treatment, including 5-phosphodiesterase inhibitors, endothelin-receptor antagonists and prostacyclin analogues, in these extremely premature born infants is still controversial. Although frequently used in current clinical practice, today, any evidence for clinical benefit of PAH-targeted medications in infants with PH-BPD is lacking.^31^ In the current study, 50% of the infants received PAH-targeted treatment. Although the proportion of patients treated with PAH-targeted therapy was higher in the surviving patients, the design of the current study does not allow any conclusion on the effects of such treatment.

The awareness that a large proportion of infants with PH-BPD die in the first 6 months after corrected age and that those who survive this period frequently experience resolution of PH is of clinical importance. In contrast to idiopathic and other forms of pulmonary arterial hypertension, PH-BPD is reversible with time and growth. Therefore, early identification of infants at risk for the development of PH-BPD is of crucial importance so that treatment strategies aiming at prevention of PH-BPD can be customised. Earlier identification could lead to a better follow-up and treatment in the first crucial months, and perhaps can increase survival. It is important to know that PH-BPD can be reversible. Also, the prospect of PH resolution in time may affect treatment and management decisions in infants with severe BPD. Finally, these observations may bring us a step closer in understanding the pathobiology of pulmonary vascular disease in preterm infants and may direct future basic research in this field.

### Limitations

This is a retrospective study with associated limitations, such as observed associations do not imply causality. In the absence of systematic screening for PH in the preterm population at 36 weeks PMA, the current study population consists of patients who were echocardiographically diagnosed PH-BPD at discretion of the treating physician and has likely resulted in a selection of more severe cases. Nevertheless, these are the cases constituting the challenges currently encountered by clinicians. Finally, the small number of infants included in the current study reduces the statistical power for the analyses of differences between survivors and non-survivors and associated risk factors.

## Conclusion

In the current study, extremely preterm born infants with PH-BPD showed a high mortality with a survival rate of less than 60% at 6 months corrected term age. Suprasystemic PAP was associated with poor outcome. Infants surviving until the corrected age of 6 months showed excellent survival and resolution of PH in almost all cases. In contrast to idiopathic and other forms of PAH, PH in this condition appears to be reversible with time and growth. However, late sequela of cardiopulmonary disease may still occur. Prospective follow-up studies should investigate whether resolution of PH in these infants can be improved by multi-modal therapies, including respiratory, nutritional and cardiovascular interventions.
